# Tumeur pancréatique rare de découverte fortuite chez un enfant en Côte d’Ivoire

**DOI:** 10.11604/pamj.2018.29.171.12392

**Published:** 2018-03-23

**Authors:** Richard Azagoh-Kouadio, Line Guei Couitchéré, Mohamed Kouyaté, Jean-Jacques Yao Atteby, Jacob Slanziahuelie Enoh, Lassina Cissé, Soumahoro Oulai

**Affiliations:** 1 Service de Pédiatrie, CHU Treichville, UFR SM Université Félix Houphouët Boigny, Abidjan, Côte d’Ivoire; 2 Service d’Anatomie et de Pathologie, CHU Treichville, UFR SM Université Félix Houphouët Boigny, Abidjan, Côte d’Ivoire

**Keywords:** Tumeur de Frantz, pancréas, enfant, chirurgie radicale, Frantz tumor, pancreas, child, radical surger

## Abstract

La tumeur pseudo-papillaire et solide du pancréas (TPPSP) est une tumeur rare. Elle touche le plus souvent la femme jeune. Décrite la première fois par Frantz en 1959, sa pathogénie demeure peu claire. C’est une tumeur de bon pronostic qui nécessite une chirurgie radicale. Les auteurs rapportent un cas de TPPSP chez une fillette de 11 ans. La symptomatologie était aiguë, faite de syndrome de compression et d’épigastralgies. L’examen trouvait une masse solide de l’hypochondre gauche. Le scanner montrait une masse de structure mixte de la queue du pancréas. Une splénopancréatectomie gauche était réalisée. Le diagnostic était confirmé par l’examen histologique avec immunohistochimie. Le suivi à long terme ne montrait pas de récidive. Le recul est de deux ans et demi. À travers cette observation et une revue de la littérature, les auteurs discutent la contribution de la radiologie dans le diagnostic et insistent sur une chirurgie radicale dans le traitement de ces tumeurs de faible degré de malignité.

## Abstract (en)

Solid pseudopapillary tumor of the pancreas (SPTP) is rare. It most often affects young women. It was first described by Frantz in 1959, but its pathogenesis remains unclear. This tumor has a good prognosis. Treatment is based on radical surgery. We here report the case of a 11-year old girl with SPTP. Symptomatology was acute, including compression syndrome and epigastralgias. Clinical examination showed a solid mass in the left hypochondrium. Scan showed mixed mass structure of the pancreas tail. Left splenopancreatectomy was performed. The diagnosis was confirmed by histological examination using immunohistochemistry. Long-term follow-up showed no recidivism. The study had a follow-up period of two and a half years. This study and literature review aimed to highlight the role of radiological examination in the diagnosis and underline the importance of radical surgery in the treatment of these tumors with a low degree of malignancy.

## Introduction

La tumeur pseudo-papillaire kystique et solide du pancréas, décrite par Frantz en 1959 [1], est une entité anatomo-clinique rare (0,3 à 2,7% des tumeurs pancréatiques) [2]. Elle se développe préférentiellement chez la jeune femme entre 25 et 30 ans, d'ethnie non caucasienne, sous la forme d'une tumeur volumineuse longtemps peu symptomatique, sans altération de l'état général. Cette tumeur est caractérisée à l'imagerie par une alternance de zones solides et kystiques nécrotico-hémorragiques. Son diagnostic est histologique [3]: fait d’une architecture papillaire avec prolifération cellulaire épithéliale monomorphe; d’atypies et de mitoses peu nombreuses, avec en immuno-histochimie, une absence, dans la majorité des cas, d'expression des marqueurs neuroendocrines. Le traitement est chirurgical et le pronostic réputé bon après exérèse complète [2]. Nous rapportons une observation avec une carcinose péritonéale chez une enfant noire africaine dont l'évolution est favorable après deux années de recul chirurgical.

## Patient et observation

Une fillette de 11 ans, d'origine noire africaine, était adressée par son pédiatre pour avis et prise en charge éventuelle d’une masse du pancréas découverte de façon fortuite sur un syndrome douloureux abdominal évoluant depuis 7 mois de façon paroxystique, accompagnées de vomissements à l’acmé des crises. Elle présente des antécédents familiaux d’un cancer du colon chez une tante. A l’examen cet enfant est en bon état général. L’examen clinique révèle une masse épigastrique, ferme, indolore, régulière, mesurant environ 7 centimètres sur la ligne joignant l’appendice xiphoïde à l’ombilic. Le reste de l’examen était strictement normal.

**Figure 1:**
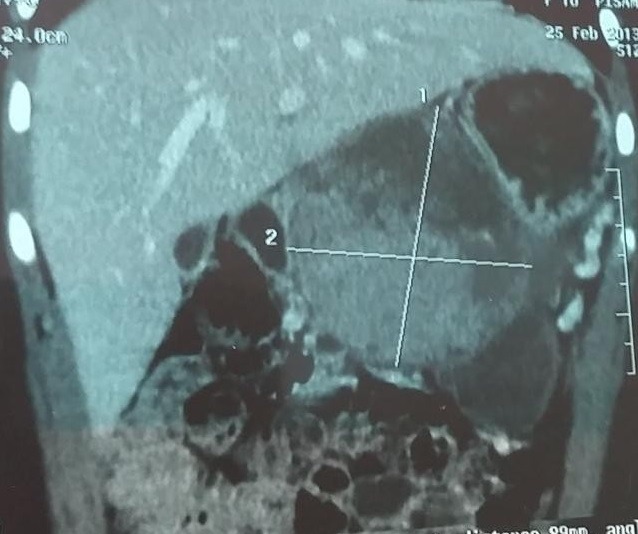
Tomodensitométrie: masse solide, encapsulée, rétropéritonéale, corporéocaudale aux dépens du corps du pancréas mesurant en axial 11.2 x 100 mm et 122 mm en hauteur

L'imagerie par échographie, tomodensitométrie (TDM) et par résonance magnétique abdominale réalisée (Figure 1) précisaient un processus tissulaire encapsulé dense, avec des zones de nécrose, vascularisé, corporéo-caudal pancréatique, mesurant 106mm de grand axe oblique en dehors, 100 mm de hauteur, 74 mm d’épaisseurs, refoulant les vaisseaux mésentériques vers la périphérie, avec réaction liquidienne péritonéal péri lésionnelle et des nodules hépatiques.

**Figure 2:**
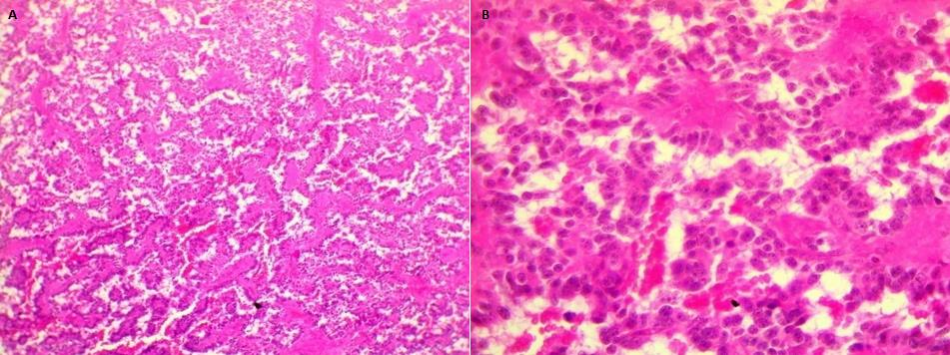
A) structures papillaires avec axe conjonctivo-vasculaire (hematoxyline-éosine x 100); B) aspect histologique: nappes de cellules monomorphes de petite taille au cytoplasme éosinophile ou vacuolaire et un noyau arrondi ou ovalaire agencées autour de fins septa fibrovasculaires (hematoxyline-éosine x 400)

Le bilan biologique était normal, sans syndrome inflammatoire ni perturbation des fonctions rénale, hépatique et pancréatique. Aucune sécrétion hormonale anormale n'était décelée. Les marqueurs tumoraux sériques (Alpha-foeto-protéine, béta-HCG plasmatique, antigène carcino-embryonnaire (ACE), CA 19,9, Œstradiol et Neuron specific enalase (NSE)) avaient des taux normaux. La biopsie écho-guidée met en évidence un aspect morphologique cadrant avec une tumeur solide pseudo-papillaire (Figure 2).

Elle a bénéficié d’une chirurgie en 2013, à l'âge de 11 ans, d'une tumeur solide pseudo-papillaire de 10 cm de grand axe avec des limites pancréatiques saines, une hypertension portale segmentaire avec absence de visualisation de la veine splénique. L'intervention chirurgicale consistait en une pancréatectomie corporéo-caudale emportant la totalité de la tumeur qui reste intacte associée à une omentectomie emportant la totalité des métastases.

**Figure 3:**
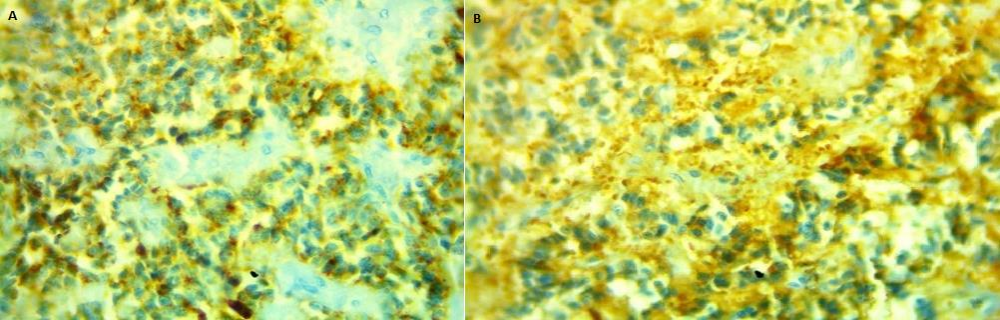
A) positivité anticorps anti-synaptophysine; B) immunomarquage positif des cellules tumorales pour anticorps anti-synaptophysine (a) et anticorps anti-chemotrypsine (b) (x 400)

L'examen anatomo-pathologique confirmait le diagnostic de tumeur pseudo-papillaire kystique et solide du pancréas (Figure 2). L’examen des prélèvements réalisés au niveau de l’épiploon ne montre pas de localisation secondaire. Néanmoins, on retrouve des remaniements fibro-inflammatoires. L’étude immunohistochimique (Figure 3) montre que les cellules tumorales expriment l’anticorps anti-synaptophysine, l’anticorps anti-chemotrypsine, le CD56, la bétacaténine, les récepteurs progestéroniques et la vimentine. Un marquage focal avec la cytokératine était noté. La chromogranine et l'ACE étaient négatifs. Les limites de résection pancréatique sont saines.

L'évolution postopératoire était simple. Aucune indication de traitement complémentaire n'était retenue. En raison du risque de récidive tumorale important, la malade était revue 1 fois tous les 3 mois: l'examen clinique, le bilan biologique et la tomodensitométrie abdominale étaient normaux.

## Discussion

Cette observation illustre la présentation classique de la tumeur pseudo-papillaire kystique et solide du pancréas sur un terrain de très rare prédilection. La tumeur pseudo-papillaire de pancréas (SPT) ou tumeur de Frantz est une tumeur rare du pancréas exocrine, ayant un faible grade de malignité. Elle survient essentiellement chez la femme jeune (de 20 à 40 ans) avec une fréquence accrue dans la population asiatique. Nous rapportons un nouveaux cas chez une enfant de 11 ans d’origine noire africaine. Les formes pédiatriques sont très rares. Notre objectif est de préciser les caractéristiques clinicopathologiques de cette tumeur rare de l’enfant, et de discuter son histogenèse.

Les manifestations cliniques ne sont pas spécifiques et sont représentées par des douleurs abdominales vagues, parfois associées à des nausées ou une masse abdominale [4,5] Cependant, la découverte fortuite de ces tumeurs est habituelle [5,6]. Plus rarement, la tumeur est révélée suite à une complication telle qu’une rupture ou une hémorragie intra-tumorale post-traumatique [5,7]. Sur le plan biologique, les enzymes pancréatiques, les marqueurs tumoraux ainsi que le bilan endocrine sont normaux [7]. A l’échographie, l’échogénicité de ces tumeurs est variable selon l’importance des zones kystiques. La tomodensitométrie (TDM) montre une volumineuse masse rétropéritonéale corporéocaudale du pancréas mesurant en axial 11.2 x 100 mm et 122 mm en hauteur. Cette masse est de densité hétérogène, solido-kystique, se rehaussant de façon modérée au niveau de sa portion charnue et est entourée par une capsule qui se rehausse surtout au temps tardif [7].

L’imagerie par résonance magnétique objective une tumeur hétérogène avec un hypersignal en T1 et T2, témoin des remaniements hémorragiques. La capsule est souvent hypointense sur les séquences en T1 [7]. L’imagerie permet également de déterminer le degré d’extension de la tumeur. La biopsie de la masse pancréatique écho-guidée montre que la tumeur est faite de prolifération de cellules monomorphe présentant une architecture très hétérogène, solide, kystique pseudo-papillaire avec présence de remaniement hémorragique. Les cellules cytoplasmes abondant clarifié sont monomorphes dépourvues d’atypie cytonucléaires. La ponction-biopsie préopératoire, à l’aiguille fine et la cytoponction sont déconseillées, lorsque le contexte ou l’imagerie évoquent une tumeur pseudo-papillaire et solide du pancréas, vu le risque d’essaimage néoplasique sur le trajet biopsique [7,8]. A la macroscopie, la tumeur est habituellement arrondie ou ovalaire et encapsulée [9]. Sa taille varie de 2,5 à 25 cm de grand axe [5], elle est le siège de remaniements nécrotiques et hémorragiques, réalisant parfois un aspect pseudo-kystique assez caractéristique [4,7]. L’effraction capsulaire avec infiltration du tissu pancréatique avoisinant est très rare [10]; celle-ci a été observée chez notre patient.

A l’histologie, la tumeur est formée de plages solides périphériques et de structures pseudo-papillaires centrales. Les cellules tumorales sont monomorphes, de petite taille et souvent agencées autour de septa fibrovasculaires [8]. Les mitoses sont habituellement très rares [4,10-12]. Il peut exister des amas d’histiocytes spumeux et des cellules géantes autour de cristaux de cholestérol [8-10]. Le stroma est habituellement de type endocrine, riche en capillaires sanguins ; les emboles vasculaires sont rares [4,10-12].

L’étude immunohistochimique est très utile, révélant une positivité diffuse pour la vimentine et la NSE dans plus de 90% des cas; l’expression de l’alpha1AT est évaluée à 50% des cas, la kératine à 70% des cas et la synaptophysine à 22% des cas [13]; le profil immunohistochimique constaté dans notre série est en accord avec les données de la littérature; l’immunomarquage pour les récepteurs d’œstrogène, et surtout de progestérone est parfois positif [8-13]; ce marquage évoque une éventuelle hormonosensibilité de la tumeur et pourrait expliquer la prédominance féminine. Dans notre série, l’expression de la progestérone était constatée dans un seul cas. Les cellules tumorales peuvent exprimer, au moins focalement, une certaine différenciation endocrine attestée par un immunomarquage positif pour certains marqueurs endocrines tels que NSE et synaptophysine [10-14]; cependant, l’immunomarquage pour la chromogranine est négatif, écartant ainsi une tumeur endocrine qui constitue le principal diagnostic différentiel [10-14]. Dans notre série, la chromogranine, constamment incluse parmi le panel d’anticorps, s’est révélée toujours négative.

Cliniquement, ces tumeurs peuvent simuler le pancréatoblastome chez l’enfant et les tumeurs endocrines kystiques, les tumeurs à cellules acineuses, et les pseudokystes inflammatoires du pancréas pour les sujets plus âgées [4-12]. Le traitement de choix est chirurgical aussi bien pour la tumeur primitive que pour les récidives et les localisations secondaires et les nodules de carcinose, vu le potentiel dégénératif de ces tumeurs [5-9].

La chirurgie est curative dans plus de 95% des tumeurs localisées [15]. La chimiothérapie, la radiothérapie et l’hormonothérapie sont rarement utilisées; leur indication est discutée particulièrement dans les formes non localisées [7]. L’évolution de ces tumeurs est habituellement favorable. La survie après une exérèse complète de la tumeur est de 80 à 90% [7]; le taux de récidive est de 10 à 15% [16]. Les formes malignes représentent environ 15% des cas [17]; les critères de malignité sont déterminés par l’envahissement vasculaire, ganglionnaire, périnerveux, des organes de voisinage, et la taille de la tumeur supérieure à 5 cm [9,10,17]. Les métastases peuvent être hépatiques, péritonéales ou pulmonaires et s’observent surtout chez le sujet âgé ; elles sont favorisées par l’effraction de la tumeur ou la biopsie de la tumeur [18].

L’étiopathogénie de ces tumeurs est controversée et discutée malgré les progrès et les apports des études immunohistochimiques et ultrastructurales. La présence de localisations extrapancréatiques permet d’évoquer deux hypothèses étiopathogéniques: soit ces tumeurs se développent à partir d’un tissu pancréatique ectopique [8], soit à partir de cellules souches totipotentes qui se différencient, au sein d’autres tissus, en cellules pancréatiques [9]. La prédominance féminine et la positivité à l’étude immunohistochimique pour les récepteurs hormonaux de la progestérone sont des arguments en faveur d’une origine embryonnaire à partir des bandelettes ovariennes gauches [7-13]. Une hypothèse d’origine centroacineuse a été également avancée, en se basant sur des constatations immunohistochimiques et ultrastructurales [19]. Cependant, ces différentes théories étiopathogéniques requièrent d’autres investigations et d’autres études pour être validées.

## Conclusion

La tumeur de Frantz est une tumeur rare du pancréas exocrine, ayant un faible grade de malignité. Elle survient essentiellement chez la femme jeune (de 20 à 40 ans) avec une fréquence accrue dans la population asiatique. Les formes pédiatriques sont plus rares. Les symptômes sont frustres et la découverte de la lésion se fait le plus souvent à l’occasion de douleurs ou lors de la palpation d’une masse abdominale. Cette tumeur est souvent bien circonscrite, associant des zones de nécrose, d’hémorragie et de prolifération cystique. Une épaisse capsule est souvent présente. Du fait de sa faible agressivité et de son caractère localisé, l’exérèse chirurgicale complète est le traitement de choix. La localisation et les éventuelles extensions locales vont déterminer la technique chirurgicale la plus appropriée. Le pronostic est très souvent favorable, surtout si la résection est passée en zone saine. Le risque de métastases, essentiellement hépatique, est faible (moins de 15%). Leur traitement est résolument chirurgical. Le risque de récidive de cette tumeur, faible, nécessite néanmoins un suivi au long cours.

## Conflits d’intérêts

Les auteurs déclarent n’avoir aucun conflit d’intérêts.
